# The peri-menopause in a woman’s life: a systemic inflammatory phase that enables later neurodegenerative disease

**DOI:** 10.1186/s12974-020-01998-9

**Published:** 2020-10-23

**Authors:** Micheline McCarthy, Ami P. Raval

**Affiliations:** 1 Peritz Scheinberg Cerebral Vascular Disease Research Laboratory, Leonard M. Miller School of Medicine, University of Miami, 1420 NW 9th Avenue, Neurology Research Building, Room # 203H, Miami, FL 33136 USA; 2 Department of Neurology, Leonard M. Miller School of Medicine, University of Miami, Miami, FL 33136 USA

**Keywords:** Alzheimer’s disease, Cerebral ischemia, Estrogen receptors, Inflammasome, Mitochondria, Menopause, Stroke

## Abstract

The peri-menopause or menopausal transition—the time period that surrounds the final years of a woman’s reproductive life—is associated with profound reproductive and hormonal changes in a woman’s body and exponentially increases a woman’s risk of cerebral ischemia and Alzheimer’s disease. Although our understanding of the exact timeline or definition of peri-menopause is limited, it is clear that there are two stages to the peri-menopause. These are the early menopausal transition, where menstrual cycles are mostly regular, with relatively few interruptions, and the late transition, where amenorrhea becomes more prolonged and lasts for at least 60 days, up to the final menstrual period. Emerging evidence is showing that peri-menopause is pro-inflammatory and disrupts estrogen-regulated neurological systems. Estrogen is a master regulator that functions through a network of estrogen receptors subtypes alpha (ER-α) and beta (ER-β). Estrogen receptor-beta has been shown to regulate a key component of the innate immune response known as the inflammasome, and it also is involved in regulation of neuronal mitochondrial function. This review will present an overview of the menopausal transition as an inflammatory event, with associated systemic and central nervous system inflammation, plus regulation of the innate immune response by ER-β-mediated mechanisms.

## Introduction

Aging is a complex, predetermined natural process. This natural process of aging is associated with reproductive senescence in most vertebrates, including mammals of both sexes. For most of the vertebrates, including lower mammals, life ends around the attainment of reproductive senescence. Among lower mammals, in only five species of whale (including killer whales) females and women experience true menopause and survive years after menopausal transition occurs. Menopause is defined as the cessation of the menstrual cycle due to anovulation. It is verified retrospectively after one year of amenorrhea [1]. The phenomenon of menopause brings multiple physiological changes in the body, and the age at which menopause occurs is increasingly recognized as an indicator for health outcomes in later life. An average age of menopause is between 45 and 51 years in the USA. The transition to menopause usually lasts about 7 years but can last as long as 14 years. A 2012 study of stroke risk in women found that if natural menopause occurs before 42 years of age, then the risk of stroke doubles [2]. In general, women’s risk of stroke and cardiac arrest increases exponentially after the onset of menopause. Both stroke and cardiac arrest cause focal and global cerebral ischemia (CI), respectively, with a major complication of cognitive decline [3–7]. Along with loss of ovarian functions, the endocrine transition from the estrogen cycling of the reproductive phase to the estrogen decline of the reproductively quiescent, post-menopausal phase is associated with mild cognitive dysfunction, which has been proposed to be a prodromal phase of Alzheimer’s disease (AD) [8]. The endocrine transition is also associated with a rise in chronic low-grade inflammation [9]. The persistent low-grade inflammation in turn accelerates ovarian failure [10]. A recent study suggested that the menopausal transition prompts an innate immune inflammatory response in the female reproductive organs that propagates to the brain, making the brain more susceptible to ischemic damage [11]. Therefore, an understanding of the biological mechanisms of menopause transition can better equip us to lower the risk of CI and AD in women and develop strategies to protect them from menopause-associated health complications.

Understanding the biological mechanisms of menopause transition or peri-menopause is complicated [12]. Systemic evaluation is difficult in humans owing to multiple life factors such as age, parity, diet, environmental factors (e.g., toxin exposures, drug abuse), genetic background, and overlapping medical comorbidities [13]. This points to the need to conduct studies using animal models of menopause; however, as routinely used laboratory rodents do not undergo menopause, we have included some discussion on how to mimic the condition of menopause in these animals in this review. Although our understanding of menopausal transition induced physiological changes is limited, it is clear that a foundational indicator of human menopause is complete ovarian failure. Menopausal women have very low circulating levels of estrogens (estrone (E_1_), 17β-estradiol (E_2_), estriol (E_3_)) and progesterone but significantly elevated follicle stimulating hormone (FSH) and luteinizing hormone (LH) levels [14, 15]. It is also well known that during the premenopausal phase of a woman’s life, estrogen confers natural protection against cerebrovascular diseases. Estrogens exert beneficial effects on a myriad of body systems, including cardiovascular, bone, and brain. It has also been shown that decline in circulating E_2_ after menopause is associated with an increased risk for cardiovascular disease, osteoporosis, cancer, diabetes, stroke, sleep disturbances, AD, and cognitive decline [8, 16]. A study investigating a cohort of healthy women transitioning into menopause showed increase in abdominal obesity, triglycerides, total cholesterol and LDL cholesterol, fasting glucose, insulin resistance, and body mass index (BMI), and increased blood pressure [17].

Numerous pharmacological strategies of the past several decades that target menopause and associated cerebro- and cardiovascular or metabolic disorders have focused on substituting or replacing lost estrogen/+progesterone. The trials focused on primary prevention—the Women’s Health Initiative (WHI)—and secondary prevention—the Heart and Estrogen/progesterone Replacement Study (HERS) and the Women’s Estrogen for Stroke Trial (WEST) [18]. These trials indicate that postmenopausal hormone therapy is not effective for reducing the risk of a recurrent stroke or death among women with established vascular disease or for prevention of a first stroke. Similar results exist for cardiovascular disease, and even recent trials of postmenopausal hormone treatment to improve cognitive outcomes have been inconsistent [19, 20]. Later trial outcomes indicate that postmenopausal hormone use may not benefit verbal cognitive function, although current and past hormone use is associated with differences in neural pathways used while assessing verbal semantic distinctions. Overall, outcomes of these trials suggested that the hormone therapy should not be initiated to prevent vascular disease among postmenopausal women. Even trials of postmenopausal hormone treatment to improve cognitive outcomes have been inconsistent. Furthermore, therapies based on estrogen substitution have been challenged by several risks associated with treatment, including heart disease, stroke, blood clots, and breast cancer.

Although estrogen disappointed in the clinic, multiple basic science studies in the field of stroke showed beneficial effects of estrogen therapy [21–24] and therefore provided better understanding of how estrogen(s) exert beneficial effects on cerebro-, cardio-, and vascular systems. It is now known that one of the key functions of estrogen is to work as a potent anti-inflammatory factor [25–27], and therefore, disturbances in the cyclic pattern of circulating estrogens at the menopausal transition activate systemic innate and adaptive immune responses [28]. The inflammasome is a key element of the innate immune response [29, 30]. The inflammasome is a multiprotein complex responsible for the activation of caspase-1 and processing of pro-inflammatory cytokines such as IL-1β and IL-18 [31, 32]. As an element of the innate immune response, the inflammasome complex is a sensor of damage associated molecular patterns (DAMPs) [33]. The onset of the innate immune responses leads to activation of the adaptive immune response, which response results in infiltration of peripheral immune cells, particularly T cell invasion of the brain [34]. Ultimately repeated or sustained activation of innate and adaptive immune responses can create the chronic low-grade inflammation typical of aging. The presence of the inflammasome complex in the cerebrospinal fluid of post-menopausal women suggests that the decline in estrogens induces a pro-inflammatory state [35]. Inflammasomes could be an important indicator of the effect of menopause on the immune system. This review assesses (1) our understanding of the menopausal transition and associated estrogen decline, (2) how these impact systemic and central nervous system (CNS) inflammatory responses, and (3) possible mechanisms by which estrogen receptor(s) regulate inflammasome activation in the brain to provide protection against ischemic damage.

## The menopausal transition process

According to the classification of the American Society for Reproductive Medicine’s Stages of Reproductive Aging Workshop (STRAW), a woman’s life is delineated into seven stages ranging from the onset of menstrual cycles at menarche and the reproductive age to the peri-menopausal and postmenopausal phases [1]. In the USA, approximately 1.3 million women become menopausal each year. The overall process of menopause transition lasts about 14 years. As mentioned in introduction, the average age of menopause is between 45 and 51 years, and the mean life span of women continues to increase beyond 80 years, which is about 5 years longer than that of men [36, 37]. Therefore, women are likely to spend at least one third of their life in the post-menopause stage, a stage that is vulnerable to the morbidities caused by immune and metabolic dysfunction and neurodegenerative disease.

An additional 1% of women experience premature menopause, before the age of 40 [38, 39]. Heredity appears to be the most important determinant of age at menopause [40]. Premature menopause due to permanent ovarian failure may be associated with sex chromosome abnormalities [39]. However, changes from the body’s natural fluctuating levels of estrogens through surgical removal of the ovaries or through natural menopause, have been independently linked to an altered immune profile, bone and blood vessel health, and changes to cognitive processes [41]. Apart from surgical removal of ovaries, cigarette smoking/tobacco use, exposure to environmental toxins, and malnutrition have been associated with premature menopause [39]. Premature menopause is defined by presence of amenorrhea, increased gonadotrophin levels, and estrogen deficiency earlier in life before actual age of menopause [42, 43]. Women with premature menopause are at higher risk of premature death, neurological diseases, psychosexual dysfunction, mood disorders, osteoporosis, ischemic heart disease, and infertility [39]. Additionally, environmental xenoestrogens, by-products of industrialization—Bisphenol A (BPA), bis(2-ethylhexyl)phthalate (DEHP), and di(n-butyl)phthalate (DBP)—modulate systemic estrogens and induce systemic and CNS inflammation [44]. Xenoestrogens (XEs) mimic or block the synthesis, metabolism, and transport of normal endogenous hormones, disturbing normal endocrine function [45]. Xenoestrogens exert their effects through estrogen receptor signaling, resulting in epigenetic changes [46]. Multiple reviews on the environmental chemical(s) have suggested that the exposure to these chemicals depletes the ovarian reserve, leading to impaired functioning of the ovary and a shortening of the reproductive lifespan [47, 48] and early menopause [49].

## Menopausal transition and medical comorbidities

Increasingly, multiple lines of experimental and public health evidence suggest that the chronic inflammation associated with estrogen decline can potentiate immune and metabolic dysfunction and neurodegenerative disease, confounding peri-menopause and posing major health challenges for twenty-first century women. Women’s risk of cardiac arrest and stroke increases exponentially after the onset of menopause. There are three main types of stroke: transient ischemic attack, ischemic stroke, and hemorrhagic stroke. It is estimated that 87 percent of strokes are ischemic. Although men have an increased stroke risk, more women than men will experience a stroke during their lifetime because of their increased life span [50, 51]. Women account for 60% of all stroke events [2, 52]. Studies consistently show that women are more functionally impaired after stroke and are less likely to receive thrombolytic therapy with tissue plasminogen activator compared with men [52]. Given the increased stroke burden and barriers to acute stroke therapy in women, it is critical to understand risk factors unique to women so that new strategies for stroke prevention can be considered. Beyond age at natural menopause, duration of ovarian activity may be a marker of stroke risk. A recent case-control study found that a longer lifetime estrogen exposure, defined as the difference between age at menopause and age at menarche, was associated with decreased stroke risk [53]. Although the underlying biological mechanisms driving increased stroke risk in women often remain unclear, they may be dependent on decline in estrogens levels around peri-menopause.

One in five women develop AD in the seventh decade of life. Late-onset AD is the most common form of dementia, and two thirds of late-onset AD patients are women. The higher longevity is one of the explanations for the late-onset AD in women; however, increasing evidence suggests that longevity alone is not the only explanation, and there may be other underlying mechanisms. Recent multi-modality brain imaging studies have compared cognitively normal 40- to 60-year-old peri-menopausal and post-menopausal women versus age- and education-matched men. The studies indicate that as women go through menopause, multiple imaging findings indicative of AD endophenotype emerge, including reduced brain glucose metabolism in frontal cortex, increased amyloid-β (Aβ) accumulation, and gray matter and white matter loss. The patterns of brain hypometabolism correlated with measured reduction in platelet mitochondrial cytochrome oxidase (COX) activity, which suggests the emergence of AD-like bioenergetic deficits in peri- and post-menopausal women [54–56]. Further studies indicate that systemic inflammation and estrogen decline associated with peri-menopause can contribute to accumulation of Aβ. Direct effects of E_2_ on neuronal Aβ have been demonstrated, showing that E_2_ decreased the generation and secretion of Aβ in primary neuronal culture and that administration of estrogen in estrogen-deprived mice reversed the elevated levels of brain Aβ [57].

As mentioned above, comorbidities such as tobacco smoking cause premature menopause and aggravated systemic inflammation. Nicotine is a potent addictive agent which inhibits aromatase enzyme activity. The aromatase catalyzes the conversion of androgens into estrogens [58]. Therefore, chronic nicotine exposure reduces circulating estrogen levels and triggers premature menopause in women [59–67]. This epidemiological finding has been modeled in female rats, in which chronic nicotine exposure reduced endogenous E_2_ levels [68]. Since E_2_ mediates its neuroprotective effects via ligand-activated estrogen receptors (ERs) subtype alpha (ER-α) and beta (ER-β), inhibition/knockdown of either of these ERs in the brain abolishes E_2_-induced ischemic protection, suggesting a key role of ER-α and/or ER-β-activation [69–71]. Estrogen receptor-β was first reported to localize to the mitochondria in 2004 [72]. Since then, it has been shown that long-term nicotine exposure selectively decreased membrane-bound and mitochondrial ER-β but not the nuclear ER-β. In a separate study, we observed that ER-β modulates inflammasome activation in the brain. However, in this study it remained to be identified which subcellular location is responsible for ER-β’s inhibitory effect on inflammasomes [68, 73–75]. Since nicotine reduces membrane-bound and mitochondrial ER-β availability and increases inflammasome activation and exacerbates post-ischemic damage in the brain of female rats, ER-β located at these two subcellular sites may be playing a role in regulation of inflammasome activation [35]. It is also likely that ER-β is translocated to cytoplasm following nicotine treatment, thus reducing the presence of ER-β at these subcellular locations and resulting in increased inflammasome activation. It has been demonstrated that ERs require palmitoylation for their transport to various subcellular sites including the plasma membrane [76–78]. Palmitoylation is a post-translational modification that regulates membrane-protein interactions and is a reversible process [79–82]. In our unpublished study in progress, nicotine reduced ER-β palmitoylation in the hippocampus. Therefore, analyzing ER-β palmitoylation levels in the subcellular fractions and investigating the role of membrane-bound ER-β with non-permeable estrogen-conjugate may provide insight into the role of membrane-bound ER-β in inflammasome activation. In an in vitro study using organotypic slice cultures, inhibition of inflammasome activation using Isoliquiritigenin (ILG) attenuated nicotine-induced ischemic cell death after oxygen-glucose deprivation [35]. Isoliquiritigenin inhibits inflammasome activation by retarding oligomerization of Apoptosis-associated speck-like protein containing a caspase recruitment domain (ASC) and NOD-like receptor-3 (NLRP3) activation. The neuroprotective effect of ILG reflects the suppression of nicotine-induced inflammasome assembly in the brain [83, 84]. Since mitochondria play pivotal roles in initiation and regulation of the NLRP3, and NLRP3 activators induce mitochondrial destabilization, NLRP3 deubiquitination, and linear ubiquitination of ASC [85], it is likely that the presence of mitochondrial ER-β may be hindering NLRP3-mitochondrial interactions. Nicotine-induced loss of mitochondrial ER-β, therefore, provides mitochondrial access to NLRP3, leading to inflammasome oligomerization and activation; however, these hypotheses remain to be tested. These findings suggest that comorbidities like environmental exposures to toxic compounds and drug addiction also need to be taken into consideration when investigating menopause-associated inflammatory responses.

## Menopause and immune responses

**Fig. 1:**
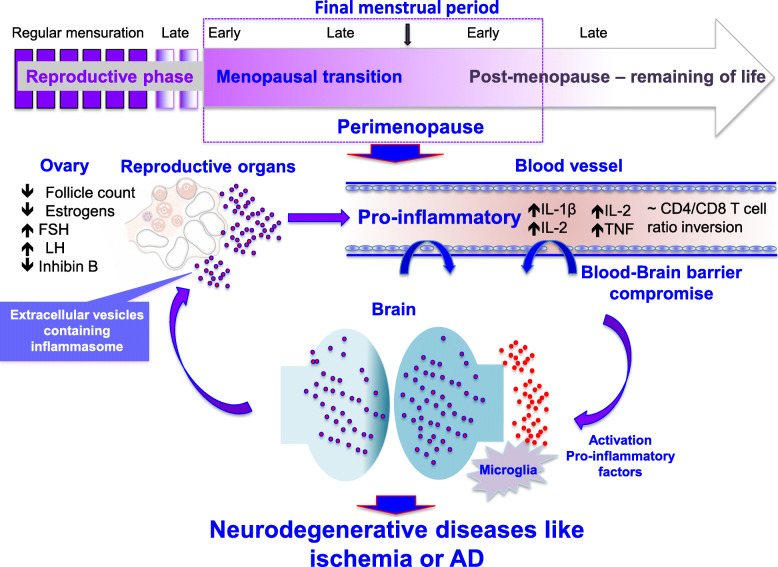
At the reproductive senescence/menopausal transition the ovarian failure is associated with release of extracellular vesicles containing inflammasomes, which may be responsible for low-grade systemic inflammation. This low-grade inflammation may compromise the blood-brain barrier (BBB), making the brain more susceptible to inflammation and neurodegenerative diseases

Estrogens are a key influence on immune and inflammatory processes, summarized graphically in Fig. 1. The role of estrogens is shown by increased inflammatory responses to infection and a higher rate of autoimmune diseases in post-menopausal women when compared to men, as well as by the variation of chronic inflammatory disease activity with the menstrual cycle, pregnancy, and menopause [86, 87]. It is very well known by now that the paucity of ovarian steroidal hormones enhances the inflammatory process predisposing menopausal women to immune disorders such as rheumatoid arthritis [88], that the pathology of multiple sclerosis worsens after menopause [89], and that post-menopausal women are more prone to stronger immune responses [90]. Additionally, studies from various laboratories have demonstrated at least a trend for increases in circulating pro-inflammatory cytokines IL-6 and TNF-alpha after natural or surgical menopause [91–94]. Deficiency of ovarian steroidal hormones potentiates the pro-inflammatory state, predisposing menopausal women to immune disorders [90, 95, 96]. The endocrine transition of the peri-menopause to the post-menopause stage, and the associated rise in chronic low-grade inflammation, is suggested to accelerate ovarian failure [10]. Declining ovarian steroidal hormones at menopausal transition coincide with higher levels of circulating interleukins IL-6, sIL-6, IL-4, IL-2, and tumor necrosis Factor (TNF) in postmenopausal women, and the latter two are also shown to reverse by hormone therapy [9, 94, 97]. Some cytokines, viz. IL-4 and IL-2 levels, are shown to increase with menopause, but the increases can be reversed by hormone therapy and are clearly suggestive of hormone dependence [9]. Synchronous to the systemic inflammatory responses, the peri-menopausal transition exhibits a decline in brain glucose metabolism and mitochondrial respiration [54, 98–100], myelin catabolism [101], and reduction of brain white matter volume [56]. These changes in systemic inflammation are also associated with beta-amyloid deposition in brain [56] and changes in neurological function [102]. Inflammatory signaling also changes T cell response, causing a reduction in CD4-positive T cell numbers in menopausal women and eventually inversion in the CD4/CD8 T cell ratio, which is indicative of aging and can be correlated with increased oxidative stress [103–105]. Overall these changes cause deterioration of the adaptive component of immune system and lead to decreased numbers of circulating B cells with menopause, especially during late menopause in comparison to peri-menopause [106]. Hormone replacement therapy retards the progress of immunosenescence by increasing the production of the CD5-B-2 subset of B cells, once more supporting the role for estrogen in the immune response [106].

## Recapitulating human female reproductive phases in laboratory animals

Estrogen’s actions are complex, and it is often challenging to systematically evaluate the biological underpinnings associated with estrogen’s actions in menopausal women. Therefore, multiple laboratories around the globe use rodent models that are invaluable tools for studying the impact of estrogen fluctuations on a variety of body systems including brain. Animal models provide researchers with opportunities to gain a fundamental understanding of the key elements underlying reproduction and aging processes, paving the way to explore novel pathways for intervention associated with known health risks. It is essential to keep in mind that some of the mechanisms associated with aging and the transition into a reproductively senescent state can differ when translating from one species to another.

The key difference between human and rodent reproductive senescence is that rodents have an estrous cycle rather than a menstrual cycle and that rodents’ uterine lining is reabsorbed rather than shed via menstruation. The estrous cycle of rat is usually 4–5 days as against the 28-day menstrual cycle in the human female. The estrous cycle is comprised of 4 main stages, viz. proestrus, estrus, metestrus, and diestrus. The transition from the diestrus to proestrus stage represents the follicular phase of the menstrual cycle, and during this transition the estrogen levels increase. The next transition from the proestrus to estrus stage is identified by a preovulatory surge of luteinizing hormone. The gradually increasing influence of progesterone corresponds to the ovulatory phase in humans. Lastly, the transition from estrus to metestrus represents the luteal phase, and this transition is associated with higher titers of circulating progesterone. The circulating ovarian hormone concentrations are lowest at the diestrus stage, and this stage correlates to the late luteal phase and menstruation in the human female [107–109]. Between the age of 9 and 12 months of age, rodents typically experience irregular estrous cycles. This phase is also known as estropause, when a persistent and prolonged estrus phase may be associated with anovulatory cycles. Eventually, animals transition into an anestrous state, where ovulatory cycles cease and low levels of gonadal steroids are evident [13, 110–112]. The fact that moderate and persistent elevations in estrogens can occur in the rat is one difference between the rodent from human peri-menopause that can complicate the interpretation of rodent models of human reproductive phases.

A review by Galea et al. reported studies showing that the naturally occurring fluctuations in ovarian hormones across the rodent estrous cycle influence hippocampal neurogenesis in adult virgin females [113]. Adult female rats have 50% more newly proliferating cells and fewer pyknotic cells in the dentate gyrus (DG) during proestrus (the high estrogen stage) compared to male rats or adult female rats in either the estrous or diestrus stage when estradiol levels are much lower [113]. These findings from ovaries-intact rodents demonstrate mitogenic and survival effects of estrogens in the hippocampal DG [113]. Studies also showed that estrous cycle and gonadal hormonal fluctuation affect the densities of dendritic spines on rodent cortical and hippocampal pyramidal neurons [114, 115]. A study also observed differential expression of genes (DEGs) in the hippocampus of rodent depending on the stage of the estrous cycle. A transcriptome analysis of the hippocampus over the course of the four consecutive stages of the estrous cycle demonstrated that sixty-seven unique genes are differentially expressed. The majority of the differentially expressed genes occur over a single stage transition: thirty-one genes were found to change from diestrus to proestrus, five genes from proestrus to estrus, and seven genes from metestrus to diestrus. An exception to this is that there are twelve differentially expressed genes with decreased expression during the proestrus to estrus transition that are also differentially expressed during the diestrus to proestrus transition [116]. Naturally, such differentially expressed genes may have correlation with the function of the hippocampus, and the hippocampus is widely believed to be essential for learning about the context in which conditioning occurs [117]. A study assessed fear conditioning procedures on naturally cycling female rats, where cued and contextual-based fear learning were tested at the same stage of the estrous cycle, during either estrus or proestrus. Female proestrus rats showed less spatial-contextual conditioning than did male or estrous female rats. These results suggest that the changes found during the proestrus part of the cycle are related to hippocampal information processing and not to general changes in learning ability, to shock sensitivity, or to state-dependent learning [118]. Another study of similar approach demonstrated that naturally cycling ovarian hormones influence fear extinction in that elevated ovarian hormone levels during the proestrus phase appear to facilitate extinction recall [119]. This study also reported sex differences in extinction recall when accounting for cycle phase in females and suggested that the elevated fear observed in female relative to male rats during extinction recall may parallel the higher prevalence of anxiety disorders in women [119]. It is now known that exposure to stressors such as foot shock during fear conditioning paradigm leads to increased expression of multiple inflammatory factors, including the pro-inflammatory cytokine interleukin-1 (IL-1) in the brain [120, 121]. IL-1 expression dependent on endogenous estrogens may be responsible for different responses to fear conditioning.

It has been shown that these hormonal fluctuations during different stages of the normal estrous cycle influence different pathological outcomes of the focal and global CI. In stroke-prone spontaneously hypertensive female rats (SHRSPs), middle cerebral artery occlusion (MCAO) during proestrus induced infarcts that were 20% smaller compared with SHRSPs in metestrus [122]. Using a similar approach in young virgin female rats, a subsequent study from our laboratory demonstrated that the increasing milieu of circulating estrogens during the transition from diestrus to the proestrus of the estrous cycle induced ischemic protection to the hippocampal CA1 neurons [123]. In the same study it was shown that increased levels of circulating estrogens during the transition from diestrus to proestrus protects the neurons from ischemic damage, by activating the cyclic AMP response element binding (CREB) protein-mediated signaling [123]. Furthermore, the study design experimentally mimicked the endogenous variations of E_2_ in ovariectomized (OVX) rats by replacing E_2_ either at 48, 72, or 96 h intervals prior to induction of an ischemic episode. In ovariectomized rats, a single bolus of exogenous E_2_ replacement 48 h prior to an ischemic episode provided maximum neuroprotection via phosphorylation of CREB protein, which requires activation of ER-β [124]. Even the long-term periodic E_2_-treatment (every 48 h) prior to global ischemia improved cognition and reduced hippocampal neuronal loss by means of ER-β activation [124]. Direct activation of ER-β by its agonist treatments every 48 h for a month improved spatial learning, memory, and ischemic neuronal survival in OVX rats, confirming role of ER-β signaling [124]. On the contrary, silencing of hippocampal ER-β using antisense approach attenuated E2-mediated ischemic protection, suggesting that ER-β plays a key role in mediating the beneficial effects of periodic E_2_ treatments [125]. In support of our study, a study by Vegeto et al. demonstrated that physiological concentrations of E_2_ pretreatment prevent brain inflammatory responses to lipopolysaccharide, a powerful inflammatory agent [126]. E_2_ treatment inhibits microglia activation, as demonstrated by the lack of acquisition of the typical reactive morphology of these cells, by the impaired expression of proteins associated with phagocytosis and cell migration, and by the reduced infiltration of leukocytes through the brain parenchyma [126]. These studies motivate investigation of prophylactic ER-β replacement regimens to reduce menopause or post-menopause-associated cognitive dysfunction, ranging from subjective symptoms (“brain fog”) to measurable post-ischemic cognitive decline.

In addition to aforementioned pretreatment strategies, studies from various laboratories using a model of global or focal ischemia in OVX rats show post-ischemic E_2_ treatment is neuroprotective [21, 23, 127]. The activation of either/both ER-α and ER-β are proven neuroprotective against ischemic insult. However, the mechanisms of neuroprotection conferred by these receptors seem to be different. Estrogen receptor-dependent mechanisms of neuroprotection could vary depending on the experimental injury model used, sex of the animal, age of the animal, pre- or post-treatment, time of E_2_ administration, type of estrogens administered, the level of estrogen administrated, and the mode of administration of the steroid (see review [128]). One of the caveats of most of the aforementioned studies, including ours, is that they were performed in young OVX female rats. Ovariectomy in the young female rat mimics the condition of surgical menopause and may lack typical physiological changes in systems and CNS that are due to natural slow decline in ovarian functions. Therefore, an approach to use naturally reproductive senescence rats could be the more appropriate model to investigate effects of menopause.

At approximately a year old, the female rat transitions into an anestrous state, where ovulatory cycles halt and low levels of gonadal steroids are present [110–112]. This situation mimics the human menopause. Utilizing the ovary-intact rat model facilitates the evaluation of the natural age-related cellular and molecular changes in brain regions involved in normal reproductive functioning and feedback. A study from Sohrabji’s laboratory using an animal model of menopause (reproductive senescence) shows that MCAO causes a larger cortical-striatal infarct in the older, ovary-intact acyclic group compared with younger females [129]. Studies from that laboratory also showed that E_2_ treatment is neuroprotective in younger females, while E_2_ paradoxically increases infarct volume in middle-aged acyclic female rats [129]. Their study also suggested that there is an age-associated loss of Insulin-Like Growth Factor-1, a neuroprotectant that decreases with advancing age and is downregulated by E_2_ treatment [129]. Consistent with the findings in menopausal women, studies have demonstrated increased pro-inflammatory cytokine levels in middle-aged female rats [130]. Elevation in inflammasome proteins has been previously reported in the hippocampus of aged rats [131]. Utilizing the ovaries-intact rat model of reproductive senescence, our recent study demonstrated the ovarian release of inflammasome-containing extracellular vesicles, which reside via blood and CSF in the CNS. This study also confirmed increased expression of inflammasome complex proteins in the CSF of peri-menopausal women, providing evidence that the observed increase in inflammation occurs in both rodents and human females [11]. Therefore, employing the ovaries-intact rat model of reproductive senescence could help understand inflammatory changes in the menopausal brain. In a recently published study, we demonstrated that adoptive transfer of serum-derived extracellular vesicles (EVs) of peri-menopausal women to young female rats triggered inflammasome activation in the rat brain, suggesting that these EVs carry the factors that can prompt inflammatory responses in the body, including CNS as they can cross the BBB [11]. It has been shown that EVs carry inflammasome proteins that play a role in the pathology of brain and spinal cord injury [132] as well as stroke [133]. It has been demonstrated that high levels of pro-inflammatory interleukin 1β exist in serum and brain of reproductively senescent female rats as compared to younger females and age matched males [11]. In an unpublished study, we observed a sex difference in systemic pro-inflammatory cytokines IL-1 and 10 profiles before and at 24 h following induction of transient MCAO; these were much higher in middle-aged female compared to male rats. These systemic inflammatory mediators released after an ischemic episode could compromise the BBB and govern the overall ischemic outcome, as middle-aged females have been shown to have severe ischemic damage as compared to young female or male counter parts.

## Estrogen receptor-beta, the inflammasome, and mitochondria

Numerous research studies, including ours, have established that E2 mediates ischemic neuroprotection through activation of estrogen receptor subtypes alpha (ER-α), beta (ER-β), and G Protein-Coupled Estrogen Receptor 1 (GPER-1; also known as GPR30) [124, 134–136]. GPER is a newly identified member of the estrogen receptor family and is shown to localize in the cerebral cortex and hippocampus, basal forebrain, thalamus, and dorsal striatum [137, 138]. Initial studies demonstrated that GPER binds with E_2_, leading to rapid activation of extracellular regulated kinases (ERKs) and cAMP generation [139–141]. Subsequently studies reported that GPER activation via G1 administration could rapidly activate PI3K-Akt and MEK-ERK, which are rapid kinase signaling pathways in the hippocampus, and exert strong neuroprotection against global cerebral ischemia (GCI) [71, 137, 142]. A recently published study shows that GPER activation upregulates interleukin-1 receptor antagonism in the hippocampus after GCI and thus limits ischemic cell death [143]. Importantly, this study suggests that GPER preserves cognitive function following GCI via enhancing the anti-inflammatory defense mechanism of neurons by upregulating interleukin-1β receptor antagonist (IL1RA) [143].

In the brain, ERα regulates reproductive neuroendocrine functions; however, ERβ plays a definitive role in a variety of neurobiological functions [144]. Utilizing subtype-selective estrogen receptor agonists has helped determine the roles for these receptors in ischemic neuroprotection. Although ER-α and ER-β share similar ligand binding domains, ER-β possesses a relative binding affinity for several steroid hormones that differs from that of ER-α [144, 145]. Propylpyrazole triol (PPT) is selective for ER-α, with a 400-fold relative binding affinity for ER-α over ER-β [146]. Diarylpropionitrile (DPN) is a subtype-selective agonist with a 70-fold greater relative binding affinity and 170-fold greater relative potency in transcription assays for ER-β than for ER-α [147, 148]. Studies have shown that both ER-α or ER-β concentration in the brain varies during aging [149, 150]. Both ER-α and ER-β decrease in the synapses of the rat hippocampal CA1 region with age but, in contrast to ER-α, the expression of ER-β is increased in response to E2 in older animals [150]. Because ER-β remains responsive to E2, understanding the mechanisms by which ER-β protects the brain from ischemic damage in reproductively senescent females could help develop future therapeutic targets. It has now been shown that periodic ER-β activation using DPN protects hippocampal neurons from ischemic cell death in reproductively senescent female rats [125]. The observed ischemic protection conferred by periodic ER-β agonist exposure reduced the inflammasome activation and decreased IL-1β proteins in the hippocampus [125]. Silencing of hippocampal ER-β using intracerebroventricular (ICV) antisense injections, increased inflammasome activation, supporting the role of ER-β in inflammasome regulation [125]. Therefore, mechanisms by which ER-β reduces post-ischemic inflammasome activation need to be further investigated.

**Fig. 2:**
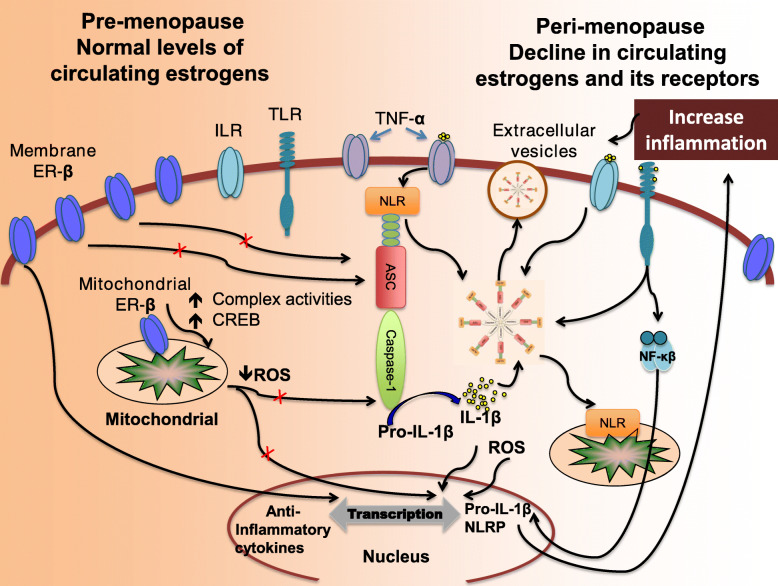
Putative mechanism of inflammasome activation in the neuron during pre- and peri-menopause. During pre-menopause, cyclic estradiol-17β(E2) maintains expression of nuclear, membrane, and mitochondrial estrogen receptor-beta (ER-β) expression, which in turn inhibits inflammasome activation by regulating mitochondrial functions, regulating biogenesis through cyclic AMP response element binding (CREB), and by reducing mitochondrial reactive oxygen species (ROS) formation. ER-β also increases expression of anti-inflammatory protein expression and reduces pro-inflammatory proteins. Decline in circulating estradiol-17β decreases estrogen receptor-beta (ER-β), causing activation of the inflammasome by reactive oxygen species (ROS). The inflammasome activates pro-caspase-1 into caspase-1, resulting in the processing of pro-IL-1β into IL-1β. Once active, IL-1β is secreted, resulting in a spread of the inflammatory response into neighboring cells. Similarly, extracellular vesicles containing inflammasome proteins get secreted, thus also contributing to the spread of the inflammatory response. ASC, Apoptosis-Associated Speck-Like Protein Containing CARD; ER-β, estrogen receptor subtype beta; ILR, interleukin receptors; IL-1β, interleukin 1β; NLR, nod-like receptor; NF-κB, nuclear factor κB; ROS, reactive oxygen species; TLR, toll-like receptors; TNFα, tumor necrosis factor alpha

It has been demonstrated that ER-β is localized and involved in regulation of mitochondrial function in neurons [73], summarized graphically in Fig. 2. Mitochondrial estrogen receptors play a direct role in estrogen-mediated preservation and regulation of mitochondrial structure and function [151–157]. The mitochondrial oxidative phosphorylation (OXPHOS) system is located in the inner mitochondrial membrane and is composed of 5 multi-subunit complexes (complexes I-V or CI-CV). Biogenesis of the mitochondrial oxidative phosphorylation system (OXPHOS) depends on both mitochondrial and nuclear genomes (see reviews [158, 159]). Estrogen receptors bind to the estrogen responsive element (EREs) located in D-loop mitochondrial DNA (mtDNA), suggesting that estrogen receptors are involved in modulation of mitochondrial gene expression [160]. In this context, a study using a human breast epithelia cell line showed that E2-stimulated increase in mRNA levels of the mtDNA-encoded genes cytochrome c oxidase subunits I and II was inhibited by ICI 182,780 (an estrogen antagonist; also known as Fulvestrant), indicating estrogen receptor dependence [155]. Another study demonstrated that cytochrome c oxidase (complex IV; CIV) subunit III mRNA levels significantly increased in the hippocampus within 3 h of E2 treatment of OVX female rats [156]. The presence of estrogen receptors in both the nuclear and mitochondrial compartments of the cell suggests regulation of mitochondrial biogenesis and function through nuclear-mitochondrial cross-talk [161, 162]. The concept of nuclear-mitochondrial cross-talk is also supported by the fact that the ER-β is provided with a mitochondrial targeting protein sequence (mTPS; aa 220-270) while ER-α lacks an mTPS [155]. In further support of the cross-talk concept, an in vitro study demonstrated shuttling of ER-β between the mitochondria and nucleus [163]. In contrast to direct regulation of mtDNA, other studies showed that mitochondrial ER-β mediates its effect through CREB phosphorylation, and, in turn, pCREB can bind directly to the D-loop of mtDNA (the control region of mtDNA) and regulates gene expression of OXPHOS subunits [164–166]. Our study showed that silencing of ER-β reduced nuclear and mitochondrial pCREB following E2 treatment, suggesting that the ER-β is essential for CREB phosphorylation (pCREB) at both subcellular locations [73]. Furthermore, silencing of ER-β lowered protein levels of mitochondria-encoded complex IV (CIV) subunits 1, 2, and 3 (Cox 1, 2, and 3), indicating the role of ER-β in pCREB-mediated mitochondrial OXPHOS protein expression [73].

The mammalian CIV is highly complex and ER-β regulates this complex in multiple possible ways that could affect oxidative phosphorylation. The CIV is a large integral membrane protein composed of several metal prosthetic sites and 13-14 protein subunits. First, subunits of this complex are partially encoded by both mitochondrial DNA and the nuclear genome [158, 159], and the assembly process of the CIV complex is very complicated and highly regulated because of dual origin. Since the silencing of ER-β lowered protein levels of mitochondria-encoded Cox 1-3, ER-β may also regulate nuclear DNA dependent subunit expression [158]. Mitochondrial ER-β mediates its effect through CREB phosphorylation, and phosphorylated CREB can bind directly to the control region of mitochondrial DNA and regulate gene expression of mitochondrial respiratory chain protein subunits [164–166]. Knockdown of ER-β reduced nuclear and mitochondrial pCREB following E_2_ treatment in rat hippocampus, which suggests ER-β is essential for CREB phosphorylation at both subcellular locations, and the mechanism by which it must be regulating CIV subunit expression [73]. Secondly, function of CIV also depends on phosphorylation of its subunits, and the CIV subunits Cox 1 and 4 undergo phosphorylation [167–169]. Thirdly, CIV subunits need correct assembly, and any defects in CIV assembly and/or stability of the enzyme result in mitochondrial dysfunction [167, 169–171]. Although it is clear that ER-β is located on mitochondria, our understanding of its presence on outer or inner mitochondrial membrane remains limited and needs investigation. Using isolated mitochondria from female brain, our study showed that CIV activity is directly regulated by ER-β and involved in regulation of glucose metabolism in the brain [73, 172].

The mitochondria are not only the cell’s powerhouses; they integrate a large number of signal transduction pathways for a wide variety of biologically active molecules. In this context, mitochondria could be considered a cellular arsenal since they (1) enclose a potent cocktail of pro-apoptotic proteins, (2) are a major site for production of reactive oxygen species, and (3) maintain calcium homeostasis. Disturbance(s) in fine-tuning of mitochondrial functions could release these “loaded weapons” thus activating cell death pathway(s). Mitochondrial dysfunction owing to ischemia triggers the generation of mitochondrial reactive oxygen species (mitoROS). Therefore, maintaining normal mitochondrial function is crucial for cell survival. Despite numerous studies conducted to understand mechanisms of mitochondrial function, there are multiple gaps, and new areas are constantly emerging which require further investigation.

Emerging studies are showing a major role of mitochondria in regulation of innate inflammation induced by inflammasome protein NLRP3, which is a sensor for disrupted homoeostasis, including perturbed mitochondrial function [173, 174]. One of the mechanisms of NLRP3 activation supported by the most studies includes the generation of mitoROS and translocation of NLRP3 to the mitochondria, leading to the release of mitochondrial DNA (mtDNA) [31, 175–177]. NLRP3 activators induce mitochondrial destabilization, NLRP3 deubiquitination, linear ubiquitination of inflammasome protein ASC, and externalization or release of mitochondria-derived molecules such as mitochondrial DNA. These molecules bind to NLRP3 that is translocated to the mitochondria and activate the NLRP3 inflammasome [85]. Mitochondria are proposed to harbor NLRP3 and be able to regulate the activity of the inflammasome complex, and mitochondrial ROS can exacerbate inflammasome immunogenic signals. In contrast, activation of mitophagy reduces inflammation by clearing mitochondrial bound NLRP3 complexes [178–180]. It is apparent that NLRP3 plays a role in ischemic pathology, as NLRP3 knockout animals have significantly reduced infarct size and neurovascular damage after focal cerebral ischemia [181]. With respect to E_2_ regulation of NLRP3 inflammasome activation, E_2_ has been reported in one study to suppress NLRP3 inflammasome gene expression in the cerebral cortex after focal cerebral ischemia [182]. Estrogen modulation of inflammation in hippocampus and of depression- and anxiety-like behavior is ER-β dependent [183]. However, the role of ER-β in activation of the NLRP3 inflammasome in the CNS remains unknown. The fact that ER-β activation confers ischemic protection, stimulates mitochondrial functions, and inhibits inflammasome activation is suggestive of its central role in cross-talk between inflammasome and mitochondria. A better understanding of the underlying mechanisms can ultimately lead to therapeutic strategies.

## Conclusion

There is increasing and compelling evidence showing that estrogen decline during the menopausal transition drives a systemic inflammatory state. This state is characterized by systemic pro-inflammatory cytokines derived from reproductive tissues, alteration in the cellular immune profile, increased availability of inflammasome proteins in the CNS, and a pro-inflammatory microenvironment which makes the brain more susceptible to ischemic and other stressors. These pro-inflammatory processes appear to compromise ER-β’s role in protecting the brain from ischemic damage and to compromise mitochondrial functions that modulate inflammasome activation. This state sets the stage for late life neurodegenerative/neurovascular disease with co-morbid cognitive dysfunction or decline. The use of ER-β-selective agonists may constitute a safer and more effective target for future therapeutic research than an ER-α agonist or E2. ER-β activation in the brain confers ischemic protection, stimulates mitochondrial functions, and inhibits inflammasome activation. ER-β agonists may be safer in that ER-β lacks the ability to stimulate the proliferation of breast or endometrial tissue. The ER-β agonist may be able to act both on the cerebro- and cardiovascular system to reduce the ischemic burden. Thus, ER-β signaling is a guide for future translational research to reduce cognitive decline and cerebral ischemia incidents and impact in post-menopausal women, while avoiding the side effects produced by chronic E2 treatment. Therefore, the model of reproductive senescence as a systemic inflammatory phase of life is crucial to understanding neurological changes that can occur in menopausal women, and to the development of novel therapeutic targets to mitigate morbidities associated with age and reproductive senescence.

## Data Availability

Not applicable

## Abbreviations

AD: Alzheimer’s disease
ASC: Apoptosis-Associated Speck-Like Protein Containing CARD
BBB: Blood brain barrier
BMI: Body mass index
CNS: Central nervous system
CI: Cerebral ischemia
CIV: mitochondrial OXPHOS system complexes I-IV
DAMPS: Damage-associated molecular patterns
DEG: Differentially expressed genes
DG: Dentate gyrus
ER-α: Estrogen receptor subtype alpha
ER-β: Estrogen receptor subtype beta
E1: Estrone
E2: 17β-estradiol
E3: Estriol
ERES: Estrogen responsive element
EV: Extracellular vesicles
FSH: Follicle stimulating hormone
GCI: Global cerebral ischemia
GPER: G protein-coupled estrogen receptor
ICV: Intracerebroventricular
ILG: Isoliquiritigenin
ILR: Interleukin receptors
IL-1β: Interleukin 1β
LH: Luteinizing hormone
MCAO: Middle cerebral artery occlusion
mtDNA: Mitochondrial DNA
mTPS: mitochondrial targeting protein sequences
NLR: Nod-like receptor
NF-κB: Nuclear factor κB
OXPHOS: Oxidative phosphorylation
ROS: Reactive oxygen species
TLR: Toll-like receptors
TNFα: Tumor necrosis factor alpha
XE: Xenoestrogens
